# Dietary Intake of Nutrients Involved in Serotonin and Melatonin Synthesis and Prenatal Maternal Sleep Quality and Affective Symptoms

**DOI:** 10.1155/2024/6611169

**Published:** 2024-07-08

**Authors:** Amber Kautz, Ying Meng, Kuan-Lin Yeh, Robin Peck, Jessica Brunner, Meghan Best, I. Diana Fernandez, Richard K. Miller, Emily S. Barrett, Susan W. Groth, Thomas G. O'Connor

**Affiliations:** ^1^ Public Health Sciences University of Rochester Medical Center, Rochester, NY, USA; ^2^ School of Nursing University of Rochester Medical Center, Rochester, NY, USA; ^3^ Clinical Research Center University of Rochester Medical Center, Rochester, NY, USA; ^4^ Obstetrics and Gynecology University of Rochester Medical Center, Rochester, NY, USA; ^5^ Psychiatry University of Rochester Medical Center, Rochester, NY, USA; ^6^ Pediatrics University of Rochester Medical Center, Rochester, NY, USA; ^7^ Pathology and Clinical Laboratory Medicine University of Rochester Medical Center, Rochester, NY, USA; ^8^ Environmental Medicine University of Rochester Medical Center, Rochester, NY, USA; ^9^ Biostatistics and Epidemiology Rutgers School of Public Health, Piscataway, NJ, USA; ^10^ Environmental and Occupational Health Sciences Institute Rutgers University, Piscataway, NJ, USA; ^11^ Neuroscience University of Rochester Medical Center, Rochester, NY, USA; ^12^ Psychology University of Rochester, Rochester, NY, USA; ^13^ Wynne Center for Family Research University of Rochester Medical Center, Rochester, NY, USA

## Abstract

Poor sleep quality and psychological distress in pregnancy are important health concerns. Serotonin and melatonin levels may underlie variation in these adverse outcomes. In this study, we examined dietary nutrients involved in serotonin and melatonin synthesis in relation to maternal sleep quality and affective symptoms during pregnancy. Pregnant women at no greater than normal medical risk at enrollment completed 24-hour dietary recalls in mid-late pregnancy. Usual intakes of vitamin B6, vitamin D, eicosapentaenoic acid (EPA) + docosahexaenoic acid (DHA), and tryptophan were estimated from dietary intake of foods and supplements using the National Cancer Institute (NCI) method. Sleep quality, depression, and anxiety were measured using validated questionnaires. Associations between nutrient intakes, sleep quality, and affective symptoms were estimated using generalized estimating equation models adjusting for potential confounding factors. In minimally adjusted models, EPA + DHA and tryptophan intakes were associated with a lower score indicating better sleep quality (*b*: −1.07, 95% CI: −2.09, −0.05) and (*b*: −12.40, 95% CI: −24.60, −0.21), respectively. EPA + DHA and tryptophan intakes were also associated with a lower odds of shorter sleep duration and sleep disturbances. In addition, tryptophan was associated with a lower odds of higher sleep latency. However, associations were attenuated and nonsignificant after adjustment for demographic and lifestyle factors. In conclusion, intakes of EPA + DHA and tryptophan were associated with improved sleep quality, but these associations were confounded by maternal demographic and lifestyle characteristics. This study highlights the need to consider dietary intake and pregnancy health in the context of demographic characteristics and lifestyle behaviors.

## 1. Introduction

Pregnant women are at increased risk of poor sleep quality and affective disorders, such as depression and anxiety [1, 2]. The prevalence of poor sleep quality has been estimated to range from 29–76% during the prenatal period [3–5]. For prenatal depression, this estimate is approximately 7–13% [6], and for prenatal anxiety approximately 18–25% [7]. These clinical concerns have been associated with increased risk of adverse maternal and neonatal outcomes, including cesarean section, postpartum depression, preterm birth, low birth weight, and intrauterine growth restriction [8–12]. Therefore, identifying predictors of sleep quality and depression and anxiety symptoms in pregnant individuals is of substantial clinical and public health relevance.

Serotonin and melatonin play a central role in sleep and mood regulation and variation in these molecules may underlie individual differences in prenatal sleep quality, depression, and anxiety [13–15]. Serotonin and melatonin are synthesized from tryptophan, an essential amino acid, along pathways requiring nutrients such as vitamin B6, vitamin D, eicosapentaenoic acid (EPA), and docosahexaenoic acid (DHA), as cofactors. Tryptophan is converted into 5 hydroxytryptophan (5-HTP) by tryptophan hydroxylase 2 (TPH2), an enzyme which is transcriptionally activated by vitamin D. 5-HTP can then be converted into serotonin, facilitated by vitamin B6. Serotonin is further metabolized into melatonin, facilitated by EPA and DHA [13, 16]. Thus, a range of nutrients contribute to serotonin and melatonin synthesis, and as a result, dietary intake may impact sleep and mood regulation in nonpregnant and pregnant individuals.

Despite the defined biological role of nutrients (including tryptophan, vitamin B6, vitamin D, and EPA/DHA) in the synthesis of serotonin and melatonin, evidence elucidating the contribution of dietary intake to synthesis pathways is limited [17]. Few studies have quantified nutrient intake from foods in relation to serotonin or melatonin synthesis. Analysis of the Nurses' Health Study cohort indicated that energy-adjusted intakes of nutrients (from food and dietary supplements) including tryptophan, omega-3 fatty acids, vitamin B6, and vitamin D, calculated from food frequency questionnaires, were not reliably associated with a primary metabolite of melatonin (6-sulfatoxymelatonin) [18]. In general, studies that have included food intake and supplementation have not found robust associations with melatonin synthesis; the inconsistency of results may depend on baseline nutrient status (e.g., deficiency) [17, 19, 20].

In contrast, more attention has been given to nutrient intake and serotonin- and melatonin-related clinical outcomes, including sleep quality, depression, and anxiety; most of these studies include community samples. The majority of evidence associating intake of these nutrients with clinical outcomes has been generated from supplementation studies [21–23]; however, nutrient intake from foods has also been considered [24, 25]. For example, dietary tryptophan intake was inversely associated with self-reported symptoms of depression, and positively associated with sleep duration among a nationally representative sample from the National Health and Nutrition Examination Study (NHANES) [24]. A meta-analysis evaluating the association between consumption of fish, a source of EPA and DHA, and depression observed a pooled risk ratio (from 26 studies) suggesting a modest protective effect (RR: 0.83, 95% CI: 0.74, 0.93) [25]. Less consideration has been given to women during pregnancy. It cannot be presumed that findings from nonpregnant individuals will generalize to pregnant individuals because of the considerable changes in maternal endocrinology, immunology, and physiology across gestation.

Previous studies of sleep quality during pregnancy have focused on nutrient biomarkers rather than dietary intake [26–30]. In regard to prenatal depression and anxiety, this is also true for vitamin D and tryptophan [26, 31]. A small number of studies have reported inverse associations between dietary intake of EPA and DHA and depression symptoms [32, 33], but have not consistently considered dietary supplements, which are widely taken and prescribed [33]. Also, to our knowledge, vitamin B6 has only been assessed in relation to depression and anxiety in the postpartum period [34, 35].

The current study was designed to address gaps in the literature by (a) assessing nutrient intake from diet, a modifiable health behavior that is a target of prenatal interventions; (b) examining both diet and supplements, which are commonly used by pregnant women but nonetheless not widely considered in studies of dietary intake [33, 36]; (c) including information on potential confounding factors, notably medication use which is often not included [32, 33]. We hypothesize that higher intakes of vitamin B6, vitamin D, EPA + DHA, and tryptophan will be associated with better sleep quality scores, and fewer depression and anxiety symptoms.

## 2. Materials and Methods

### 2.1. Study Population

The current analysis is based on data from a prospective longitudinal cohort study of prenatal influences on child health outcomes in Rochester, NY [37]; the cohort is part of the NIH Environmental Influences on Child Health Outcomes program [38]. Between December 2015 and April 2019, *n* = 326 women were recruited in their first trimester of pregnancy from outpatient obstetric clinics affiliated with the University of Rochester. Eligibility criteria were age 18 years or older, singleton pregnancy, no known substance abuse problems or a history of psychotic illness, ability to communicate in English, and not greater than normal medical risk. Women with significant endocrine disorders (e.g., polycystic ovary syndrome) or obstetric problems were excluded. This analysis includes women who provided dietary data and completed questionnaires on sleep quality and depression or anxiety symptoms in mid-late pregnancy. The University of Rochester School of Medicine and Dentistry Institutional Review Board (RSRB00058456) approved the study protocols; all participants provided written informed consent and were compensated for participation.

### 2.2. Dietary Intake

Participants were asked to complete at least two 24-hour dietary recalls during mid-late pregnancy. Structured dietary recall interviews were conducted over the telephone by a trained nutritionist, using the United States Department of Agriculture's (USDA's) automated multiple pass method [39, 40]. Participants were asked to recall all foods, beverages, and dietary supplements consumed in the 24 hours prior to the interview. Dietary data were processed using the Nutrition Data System for Research (NDSR) software and supplement data were assessed using the Dietary Supplement Assessment Module (DSAM), from the University of Minnesota (Nutrition Coordinating Center, University of Minnesota, Minneapolis, MN, 2017). The National Cancer Institute (NCI) method (which adjusts for within-person measurement error) was used to estimate usual intake of the target nutrients [41–45]. Covariates used in the NCI method were chosen *a priori* and included maternal age, race and ethnicity, education, early pregnancy body mass index (BMI), average gestational age at the time of dietary data collection, and day of dietary data collection (weekend/weekday). Estimates were generated to be isocaloric and reflected intake per 1000 calories consumed [46]. As prenatal supplements contributed to the daily intake of vitamin B6, vitamin D, and EPA/DHA, dietary supplement values were added to the NCI estimates [47]. Prenatal dietary intakes of the target nutrients are reported in two ways: (1) food only; (2) food plus dietary supplements. As the NCI method does not allow for estimation of usual individual intake, median values were derived from raw 24-hour dietary recalls, where participants with >1 recall had an average across recalls taken. At least one 24-hour dietary recall was collected for 253 participants in trimester 2 (of which 172 (68%) had two recalls), and for 74 participants in trimester 3 (of which 37 (50%) had two recalls), for a total of 270 participants with at least one 24-hour recall collected.

### 2.3. Sleep Quality and Affective Symptoms

Symptoms of depression and anxiety were measured in each trimester and sleep quality was assessed in trimesters 2 and 3. To optimize temporality between nutritional exposures and outcomes, we focused on outcome measures collected at time points consistent with the nutrition variables (trimesters 2 and 3). Sleep quality was assessed using the Pittsburgh Sleep Quality Index (PSQI) [48]. The 19-item PSQI assesses 7 components of sleep quality; global scores range from 0 to 21, and component scores from 0 to 3 with higher scores indicative of worse sleep quality. Depression symptoms were assessed using the Edinburgh Postnatal Depression Scale (EPDS) [49]. The EPDS is a 10-question self-rated scale used to identify women at risk for perinatal depression. Overall scores range from 0 to 30, with higher scores indicating more severe depression symptoms. Anxiety symptoms were measured using the Penn State Worry Questionnaire (PSWQ) [50]. The PSWQ is a 16-item questionnaire, with total scores ranging from 16 to 80 and higher scores indicating greater worry.

### 2.4. Covariates

Participant demographics and lifestyle characteristics were collected via questionnaires at enrollment and at each trimester visit. Covariates considered for inclusion in this analysis were selected *a priori* based on previous literature, and included maternal age, race and ethnicity, parity, early pregnancy BMI, highest level of education achieved, physical activity, and smoking status during pregnancy [26, 28, 32]. As use of medications intended to improve sleep quality and affective symptoms is likely to be associated with these outcomes, self-reported medication use in trimesters concurrent with outcome ascertainment were also considered. Specifically, selective serotonin reuptake inhibitors (SSRIs) and other psychiatric medications were added to models of affective symptoms, and sleeping medications were added to models of PSQI score. Dichotomous variables indicating any use in each trimester of pregnancy vs. no use were created for each category. Recognizing that race and ethnicity is a social construct that impacts diet as well as sleep and stress outcomes, race and ethnicity was categorized into four groups, including Non-Hispanic White, Non-Hispanic Black, Hispanic, and Other race and ethnicity. Parity was categorized as nulliparous or multiparous. Early pregnancy BMI, a widely used proxy for prepregnancy BMI, was calculated using the earliest weight measurements recorded in the electronic health record during the first trimester of pregnancy combined with self-reported height. To characterize highest level of educational attainment, four categories were created: high school, some college, bachelor's degree, and postgraduate degree. Physical activity was measured using the Pregnancy Physical Activity Questionnaire (PPAQ), which quantified physical activity during pregnancy [51]. Moderate to vigorous physical activity (MVPA) was calculated based on PPAQ responses in the second and third trimesters and was measured in metabolic equivalents of task (MET) hours per week. Smoking status was categorized according to whether the participant had ever smoked during the current pregnancy (yes/no). Among participants with both dietary and outcome data available, missing values among covariates did not exceed 3.1%, thus complete case analysis was conducted.

### 2.5. Statistical Analysis

Descriptive statistics were used to summarize participant characteristics. The distributions of NCI estimated intakes of vitamin B6, vitamin D, and EPA + DHA were not normal after adding the supplement values and were therefore natural log transformed. Age was standardized and early pregnancy BMI was standardized and natural log transformed. Physical activity was natural log transformed. To facilitate model convergence, education was further categorized into a binary variable indicating high school completion at most vs. more than high school completion.

Generalized estimating equation (GEE) models were constructed to assess the relationship between nutrients, sleep quality, and affective symptom outcomes. In this analysis, models were separately constructed to assess the influence of nutrient intake on the three outcomes: (1) sleep quality using the global PSQI score (0–21); (2) depression symptoms using the EPDS score (0–30); and (3) anxiety symptoms using the PSWQ score (16–80). The standard errors for the coefficients were estimated using 200 bootstrapped datasets generated from sampling with replacement.

For each nutrient-outcome relationship, we fit minimally adjusted and extended models. Minimally adjusted models included only trimester and extended models additionally included nutrient-specific variables and demographic and medical variables identified above. In extended models for vitamin D, EPA + DHA, and tryptophan, intakes of macronutrients were adjusted because they could influence the absorption or bioavailability of the micronutrient exposures. Percent of calories from fat was adjusted in analyses with vitamin D and EPA + DHA. Percent of calories from carbohydrate and protein were adjusted in analyses with tryptophan. Lifestyle and demographic covariates were adjusted in all extended models, including maternal age, race and ethnicity, parity, early pregnancy BMI, education, moderate to vigorous physical activity, and smoking during pregnancy [26, 28, 32]. Specific maternal self-reported medication use for individual affective symptom outcomes was added to extended models. This included the addition of a “sleeping medication” variable in PSQI models that was derived from PSQI question 7 that asked about the frequency of sleeping medication use in the past 30 days. In EPDS and PSWQ models, variables were adjusted to indicate reported use (yes/no) during the second and third trimesters of SSRIs or other psychiatric medications.

Post hoc analyses were also conducted to help understand which covariates were responsible for observed changes in effects between minimally adjusted and extended models. Models for which dietary estimates changed from significant to nonsignificant in the adjusted model were reanalyzed; each covariate was individually removed from the model to assess the percent change in the effect estimate associated with its removal (e.g., age was removed, and the effect estimate assessed, and then age was added back in and education was removed). Individual covariates resulting in a >10% strengthening in the beta coefficient upon their removal were grouped and simultaneously removed from the extended model. Notably, bootstrapping was not implemented to generate 95% confidence intervals in post hoc analyses as only changes in the effect estimate were of interest.

Lastly, associations between PSQI component scores (ranging from 0 to 3, with higher scores indicating worse sleep quality), and nutrients significantly associated with global PSQI scores in at least minimally adjusted models were assessed using GEE models. Statistical significance for all analyses was defined by *p* < 0.05. To account for multiple comparisons of the combined analyses with four nutrient exposures and three sleep and affective symptom outcomes, the Bonferroni-adjusted *p* value (0.004) was used to indicate significance. Conventional significance criteria at *p* < 0.05 was considered as nominal significance. All analyses were conducted using SAS 9.4 (SAS Institute, Cary, NC).

## 3. Results

### 3.1. Maternal Characteristics and Dietary Intake

Distributions of maternal demographic and lifestyle characteristics are presented in Table 1. The mean age at enrollment was 29 years. Median early pregnancy BMI was 26.3 kg/m^2^, and 54.4% of participants were overweight or obese in early pregnancy. A majority of women were Non-Hispanic White (57.8%), had an education level exceeding high school (64.8%), and were multiparous (64.4%). Median moderate to vigorous physical activity levels were 118.65 MET hours/week in trimester 2 and 126.18 MET hours/week in trimester 3, and most women did not smoke during pregnancy (93.1%). Compared to participants who did not provide prenatal dietary data, those who did were older and had a lower early pregnancy BMI (Supplementary Table 1).

The medians of total intakes of nutrients from food only for trimester 2 were 1.89 mg/day, 4.03 mcg/day, 0.04 grams/day, and 0.97 grams/day for vitamin B6, vitamin D, EPA + DHA, and tryptophan, respectively; for trimester 3, values were 1.85 mg/day, 4.23 mcg/day, 0.03 grams/day, and 0.92 grams/day, for vitamin B6, vitamin D, EPA + DHA, and tryptophan, respectively. As anticipated, dietary supplement intake greatly increased intake estimates of vitamin B6, vitamin D, and EPA + DHA. The medians of total intakes of nutrients after inclusion of supplement data for trimester 2 were 4.53 mg/day, 14.23 mcg/day, and 0.08 grams/day for vitamin B6, vitamin D, and EPA + DHA, respectively; for trimester 3, values were 4.39 mg/day, 13.18 mcg/day, and 0.06 grams/day for vitamin B6, vitamin D, and EPA + DHA, respectively. Supplementary Figure 1 indicates the number of cohort participants who provided prenatal dietary data as well as responded to questionnaires of sleep quality, depression, and anxiety symptoms.

### 3.2. Nutrient Intake and Sleep Quality, Depression, and Anxiety Symptoms

**Figure 1:**
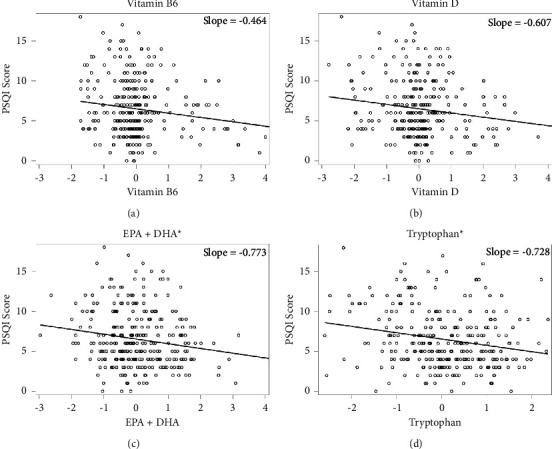
Minimally adjusted associations between nutrients involved in serotonin and melatonin synthesis and Pittsburgh sleep quality index (PSQI). Minimally adjusted models only adjust for trimester of dietary intake and sleep quality measurement. Panel (a) above indicates the minimally adjusted association between vitamin B6 and PSQI score, where vitamin B6 intake amounts are natural log transformed mg/1000 kcals; panel (b) indicates the association between vitamin D and PSQI score, where vitamin D intake amounts are natural log transformed mcg/1000 kcals; panel (c) indicates the association between EPA + DHA and PSQI score, where EPA + DHA intake amounts are natural log transformed grams/1000 kcals for EPA + DHA; and panel (d) indicates the association between tryptophan and PSQI score, where tryptophan intake amounts are grams/1000 kcals. To allow for comparison of slopes across nutrients, intake amounts of each nutrient were standardized (i.e., slopes presented in this figure were generated from standardized nutrient amounts and differ from those presented in Table 2). ^*∗*^*p* < 0.05.

In minimally adjusted GEE models, higher intake of EPA + DHA (*b*: −1.07, 95% CI: −2.09, −0.05) and tryptophan (*b*: −12.40, 95% CI: −24.60, −0.21) were associated with lower (better) sleep quality scores (*p* < 0.05) (Table 2; Figure 1). However, when models were adjusted for macronutrient intake, demographic and lifestyle factors, and sleeping medication use, the estimates were attenuated (Table 2). Post hoc analyses indicated positive confounding of the EPA/DHA and PSQI association by physical activity and sleeping medication use, and of the tryptophan and PSQI association by carbohydrate and protein intake, age, race and ethnicity, parity, smoking during pregnancy, and early pregnancy BMI (Supplementary Table 2).

As EPA + DHA and tryptophan intake were associated with global PSQI score in minimally adjusted models, associations between these nutrients and the seven PSQI component scores were further assessed (Table 3). The sleep latency component corresponds to the amount of time on average taken to fall asleep in the past month. In the minimally adjusted model, higher tryptophan intake was associated with a lower odds of scoring higher (taking longer to fall asleep) on the sleep latency component (OR: 0.95, 95% CI: 0.90, 0.99). The sleep duration component corresponds to the self-reported number of hours slept per night. In minimally adjusted models, higher EPA + DHA intake (OR: 0.68, 95% CI: 0.47, 0.97) and higher tryptophan intake (OR: 0.94, 95% CI: 0.89, 1.00) was associated with a lower odds of scoring higher (sleeping less) on the sleep duration component. The sleep disturbances' component corresponds to the quantity of times a participant had trouble sleeping, where a higher score indicates an increased frequency. In minimally adjusted models, higher EPA + DHA and tryptophan intake was associated with a lower odds of sleep disturbances (OR: 0.58, 95% CI: 0.43, 0.80) and (OR: 0.91, 95% CI: 0.86, 0.96). These associations were not maintained when demographic and lifestyle factors were included in the model (Table 3). Post hoc analyses did not identify any single variable that was responsible for a 10% change in the effect estimate upon removal from the extended model, indicating that attenuation of effects from the minimally adjusted to the extended models was likely attributable to a combination of covariates (Supplementary Tables 3–5).

Results in Table 2 indicate that there was not a statistically reliable association between nutrients involved in serotonin and melatonin synthesis and depression or anxiety symptoms. Effect sizes from extended models were in the expected direction, for all nutrients aside from tryptophan, and ranged from −3.22 (PSWQ) to −0.65 (EPDS) for vitamin B6; −0.95 (PSWQ) to −0.78 (EPDS) for vitamin D; −2.45 (PSWQ) to −1.48 (EPDS) for EPA + DHA; and 5.88 (PSWQ) to 23.83 (EPDS) for tryptophan.

## 4. Discussion

We examined dietary intake of nutrients involved in the synthesis of serotonin and melatonin in relation to sleep quality and affective symptoms in a diverse, generally healthy pregnancy cohort. Increased intake of EPA + DHA and tryptophan were associated with better sleep quality, a lower odds of shorter sleep duration, and a lower odds of more sleep disturbances; and increased tryptophan intake was also associated with a lower odds of greater sleep latency in minimally adjusted models. However, these associations were reduced and not statistically significant when maternal demographic and lifestyle characteristics were included in the model. There was no evidence that dietary intake was associated with affective symptoms throughout pregnancy. These findings provide novel data to support the multifactorial etiology of sleep quality and affective symptoms in pregnancy.

To our knowledge, dietary intakes of vitamin B6, vitamin D, EPA/DHA, and tryptophan have not been assessed in relation to sleep outcomes during pregnancy. The assessment of dietary intake is an important complement to studies of nutrient biomarkers—which have been previously studied in this context—because of the emphasis placed on diet as a modifiable health behavior and the focus on diet-based changes as an intervention target [52]. Several bivariate associations of at least modest magnitude were found with sleep quality measures, but in each case, the effect could be explained by maternal demographic and lifestyle factors. Previously, red blood cell DHA concentrations have been associated with better sleep during pregnancy [27]. However, potentially relevant confounders, including physical activity and sleeping medication use, were not considered. Our dietary assessments support and extend these prior results, but also underscore that dietary intake is likely confounded by maternal factors that are associated with sleep quality.

Tryptophan, an essential amino acid, has a clear mechanistic pathway influencing sleep; nonetheless, nutrition-based measures may not adequately capture bioavailability and/or synthesis well enough to predict clinical outcomes. Several metabolic factors influence tryptophan's availability for serotonin and melatonin synthesis [13], and these may be difficult to ascertain from dietary assessments. We considered this and adjusted for macronutrient intake likely to influence tryptophan's transport across the blood brain barrier. However, it is possible that a more specific measure of the large neutral amino acids (e.g., intake of tyrosine, isoleucine, leucine, valine, and phenylalanine) that compete with tryptophan for transport may be a better measure.

Depression and anxiety symptoms in the prenatal and postnatal period are common, affecting approximately 15% of the population. There is considerable interest in the possibility that modifiable behaviors such as diet may be one route for promoting maternal and child health during pregnancy. There is limited research within the emerging field of nutritional psychiatry using dietary assessments in the prenatal period; much of the focus to date is on biomarkers and the postpartum period [31, 35, 53, 54]. In the current study, we did not observe reliable associations between nutrients involved in serotonin and melatonin synthesis and prenatal affective symptoms. That is inconsistent with some prior evidence, particularly concerning EPA and DHA. Data from the ALSPAC cohort [32] indicated that relative to women consuming more than 1.5 grams of omega-3 fatty acids from seafood per week, those who consumed none had 1.54 (95% CI: 1.25, 1.89) times the odds of having high levels of depression symptoms at 32 weeks gestation. Notably, only 2% of the study population was taking an omega-3 supplement. In addition, an observational study among pregnant women in Japan showed that higher intakes of EPA and DHA were associated with a lower prevalence of depression symptoms, but information regarding dietary supplement intake was not considered [33]. Lack of information on dietary supplements, particularly in studies of women residing in the US, may be an important limitation given the prevalence of supplement use and generally low level of seafood intake in most American diets [55]. It is also possible that dietary intake of EPA and DHA was not high enough in our sample to have an effect on affective symptoms. Results from previous randomized placebo-controlled trials of omega-3 fatty acids for perinatal depression symptoms have been inconsistent [56], although observational studies reporting statistically significant associations between dietary intake of omega-3 fatty acids and depression symptoms during pregnancy included participants with substantially higher intakes [32, 33]. The median intake of EPA and DHA in our population, even after including supplements, was only 0.06–0.08 grams/day in mid-late pregnancy, which is similar to estimated intake among a nationally representative sample of women during pregnancy [57]. Notably, intakes of other target nutrients in our sample, including tryptophan [24], vitamin B6 [58], and vitamin D [58], were also similar to usual intake estimates among women in NHANES.

Research findings on affective symptoms and other nutritional or dietary markers involved in serotonin and melatonin synthesis is much more limited. Similar to our results, one study reported no association between plasma tryptophan in mid-gestation and depression symptoms [26]. We are unaware of prior studies that have examined the relationship between vitamin B6 and affective symptoms, although the role of vitamin B6 in modifying the effect of neurotransmitters such as serotonin has been demonstrated [59]. Several previous studies have reported that lower concentrations of vitamin D may be associated with depression symptoms during pregnancy [31, 60, 61]. In contrast to these studies which used biomarker evidence, we observed no association between depression symptoms and dietary recall of vitamin D intake. This further example that prior research on biomarker assessment is not replicated with nutritional assessment is an important observation, particularly for the methodological lessons it provides for the emerging field of nutritional psychiatry and clinical and experimental research on prenatal influences on maternal and child health.

The current study is one of few that quantifies dietary intake of nutrients involved in serotonin and melatonin synthesis, from both foods and supplements, among pregnant women, in relation to sleep quality and depression and anxiety symptoms. Our findings of no association between dietary intake of these nutrients and sleep quality or depression and anxiety symptoms, independent of lifestyle and demographic factors, underscores the importance of reproducing positive findings from biomarker studies as well as from studies in populations with drastically different nutrient intakes to assess the generalizability of findings prior to developing recommendations. Here, our findings indicate that interventions aimed at improving sleep quality among women during pregnancy should be multifactorial and focus on diet in combination with other lifestyle factors including physical activity. Notable strengths of the current study include use of the National Cancer Institute (NCI) method for estimating associations between dietary intake measured with 24-hour dietary recalls and sleep and affective symptoms; rigorous longitudinal data collection to allow for extensive consideration of potential confounding factors (e.g., medication use); and the inclusion of nutrient intakes from dietary supplements, in addition to intake from food sources, to estimate an overall average intake value.

Several limitations need to be considered when interpreting our results. First, the sample size for this study was relatively smaller than prior studies [32, 33]. In addition, dietary data, which are prone to both recall and social desirability bias, were self-reported by participants. However, we used “gold standard” dietary collection techniques (i.e., the USDAs multiple pass method) to minimize error from self-report. We must also acknowledge that three recalls are recommended to estimate usual intake most accurately [62]. One- or two-days of recalls, like the majority of our participants contributed, do not capture random within-person variability in dietary intake necessary to represent usual intake over long periods of time. Since this error is random, the mean dietary intakes are close to the true mean, but the standard deviations are overestimated, resulting in attenuated effect estimates. To overcome this limitation, we applied the NCI method to model random within-person variability in our data. Also, we did not assess nutritional biomarkers in this cohort, although few biomarkers exist that accurately reflect long-term dietary intake, nutrient status (e.g., deficiency) may show moderate associations between dietary exposures and sleep quality or depression and anxiety symptom outcomes. In addition, we observed low intakes of some nutrients (especially EPA and DHA), relative to other studies [32, 33], which may have restricted our ability to detect an association. Finally, our sample represents women who were at no greater than normal medical risk at enrollment, which is not representative of all women entering pregnancy. In light of the aforementioned limitations, a number of considerations are necessary before translating these findings to other populations, including baseline nutritional status (i.e., biomarker concentrations), average population nutrient intakes (from foods and dietary supplements), and overall medical risk of women entering pregnancy. Future studies designed to accurately estimate usual diet are necessary to elucidate the role that nutrients, including the interaction between dietary intake and biomarker concentrations, play in sleep quality as well as anxiety and depression symptoms during pregnancy.

## 5. Conclusions

Studies that consider both nutrient intake from foods and supplements in the prenatal period particularly for vitamin B6, vitamin D, and tryptophan, in relation to sleep quality and depression and anxiety symptoms are lacking. Filling that gap, in the current study, despite an observed association between EPA + DHA and tryptophan intake and sleep quality in minimally adjusted models, associations were confounded by additional nutrient, demographic, and lifestyle characteristics. This highlights the importance of considering nutrient intake, alongside other potential contributing factors, when assessing the etiology of sleep quality symptoms during pregnancy.

## Figures and Tables

**Table 1:** Characteristics of study participants with prenatal dietary data available.

	Sample size	Value
Race and ethnicity, *N* (%)	270	
NH white		156 (57.78)
NH black		63 (23.33)
Hispanic		29 (10.74)
Others		22 (8.15)
Education, *N* (%)	270	
≤ High school		95 (35.19)
Some college		39 (14.44)
Bachelor's degree		68 (25.19)
Postgraduate degree		68 (25.19)
Parity, *N* (%)	270	
Nulliparous		96 (35.56)
Smoking during pregnancy, *N* (%)	261	
Yes		18 (6.90)
Trimester 2 medication use, *N* (%)		
Sleeping medication	237	33 (13.9)
SSRI	245	11 (4.49)
Psychiatric, not SSRI	245	4 (1.63)
Trimester 3 medication use, *N* (%)		
Sleeping medication	67	11 (16.4)
SSRI	71	4 (5.63)
Psychiatric, not SSRI	71	1 (1.41)
Age in years, mean (SD)	270	29.17 (4.56)
Trimester 2 physical activity, median (25th and 75th)	245	
MVPA met hours/week		118.65 (70.53, 200.55)
Trimester 3 physical activity, median (25th and 75th)	71	
MVPA met hours/week		126.18 (65.10, 258.83)
Early pregnancy BMI, median (25th and 75th)	270	26.26 (22.86, 32.28)

SSRI = selective serotonin reuptake inhibitor, MVPA = moderate to vigorous physical activity, and BMI = body mass index.

**Table 2:** Associations between nutrients involved in serotonin and melatonin synthesis and sleep quality and depression and anxiety symptoms during pregnancy.

	Minimally adjusted^2^ beta coefficient (95% CI)^4^	Extended^3^ beta coefficient (95% CI)^4^
Sleep quality (PSQI)	(*N* = 242)	(*N* = 238)

Vitamin B6^1^	−0.75 (−2.72, 1.23)	0.04 (−4.78, 4.87)
Vitamin D^1^	−0.95 (−1.90, 0.01)	−0.48 (−3.24, 2.28)
EPA + DHA^1^	−1.07 (−2.09, −0.05)	−0.92 (−3.57, 1.72)
Tryptophan^1^	−12.40 (−24.60, −0.21)	0.22 (−40.44, 40.88)

Depression (EPDS)	(*N* = 261)	(*N* = 253)

Vitamin B6	−0.77 (−2.99, 1.45)	−0.65 (−3.89, 2.58)
Vitamin D	−1.05 (−2.74, 0.64)	−0.78 (−3.24, 1.68)
EPA + DHA	−0.69 (−2.11, 0.73)	−1.48 (−3.76, 0.81)
Tryptophan	−10.49 (−29.72, 8.75)	23.83 (−9.25, 56.90)

Anxiety (PSWQ)	(*N* = 259)	(*N* = 251)

Vitamin B6	−2.44 (−6.39, 1.52)	−3.22 (−11.67, 5.24)
Vitamin D	−0.99 (−5.13, 3.15)	−0.95 (−8.37, 6.47)
EPA + DHA	−0.77 (−3.70, 2.16)	−2.45 (−8.39, 3.49)
Tryptophan	−11.37 (−49.44, 26.71)	5.88 (−100.55, 112.32)

^1^Units are natural log transformed Mg/1000 kcals for vitamin B6, natural log transformed Mcg/1000 kcals for vitamin D, natural log transformed grams/1000 kcals for EPA + DHA, and grams/1000 kcals for tryptophan. ^2^Minimally adjusted models are adjusted for trimester. ^3^Variables considered in extended models differ by exposure and outcome. All included trimester, maternal age, early pregnancy BMI, race and ethnicity, education, parity, physical activity, and smoking during pregnancy, as well as medication use, specifically sleeping medication use in PSQI models and SSRI and other psychiatric medication use in EPDS and PSWQ models. In addition, extended vitamin D and EPA + DHA models included the percent of calories consumed from fat, and extended tryptophan models included the percent of calories consumed from protein and from carbohydrate. ^4^Standard errors and corresponding 95% confidence intervals were generated using 200 bootstrapped datasets. PSQI = Pittsburgh sleep quality index, EPA + DHA = eicosapentaenoic acid and docosahexaenoic acid, EPDS = Edinburgh Postnatal Depression Scale, and PSWQ = Penn State Worry Questionnaire.

**Table 3:** Associations between EPA + DHA^1^ and tryptophan^2^ intake and Pittsburgh sleep quality index (PSQI) component scores during pregnancy^3^.

PSQI component scores	EPA + DHA		Tryptophan	
	Minimally adjusted^4^ odds ratio (95% CI)	Extended^5^ odds ratio (95% CI)	Minimally adjusted^4^ odds ratio (95% CI)	Extended^5^ odds ratio (95% CI)
Subjective sleep quality	0.74 (0.52, 1.04)	0.93 (0.65, 1.34)	0.94 (0.89, 1.00)	1.01 (0.90, 1.13)
Sleep latency	0.85 (0.64, 1.13)	0.95 (0.70, 1.30)	**0.95 (0.90, 0.99)**	0.91 (0.81, 1.02)
Sleep duration	**0.68 (0.47, 0.97)**	0.83 (0.54, 1.29)	**0.94 (0.89, 1.00)**	1.00 (0.88, 1.13)
Habitual sleep efficiency	0.76 (0.56, 1.02)	0.80 (0.55, 1.16)	0.97 (0.92, 1.01)	0.98 (0.89, 1.09)
Sleep disturbances	**0.58 (0.43, 0.80)**	0.79 (0.55, 1.14)	**0.91 (0.86, 0.96)**	0.93 (0.83, 1.05)
Use of sleep medication	0.92 (0.57, 1.47)	0.72 (0.39, 1.32)	1.02 (0.94, 1.10)	1.10 (0.91, 1.34)
Daytime dysfunction	0.84 (0.61, 1.16)	0.87 (0.58, 1.29)	0.97 (0.93, 1.02)	1.00 (0.88, 1.14)

^1^EPA + DHA units are natural log transformed grams/1000 kcals. ^2^Tryptophan units are 10 mg/1000 kcals. ^3^Sample sizes varied by component scores. For minimally adjusted models, *N* = 252 for sleep disturbances and 253 for all other components. For extended models, *N* = 242 for sleep latency and sleep disturbances, *N* = 243 for subjective sleep quality, sleep duration, and habitual sleep efficiency, *N* = 247 for use of sleep medication, and *N* = 246 for daytime dysfunction. ^4^Minimally adjusted models include only trimester. ^5^Extended EPA + DHA models included trimester + percent of calories consumed from fat, maternal age, early pregnancy BMI, race and ethnicity, education, parity, physical activity, smoking during pregnancy, and sleeping medication use (yes/no), with the exception of the extended model for use of sleep medication, where sleeping medication use was modeled as the outcome and was not considered as a covariate. Extended tryptophan models included the same covariates as EPA + DHA models with the exception of adjusting for percent of calories from carbohydrate and protein intake rather than fat intake. EPA + DHA = eicosapentaenoic acid + docosahexaenoic acid and PSQI = Pittsburgh sleep quality index. The significance of all bold values is *p* < 0.05.

## Data Availability

Data described in the manuscript can be made available through the Environmental Influences on Child Health Outcomes (ECHO) program.

## Supplementary Materials

Supplementary Materials Supplementary Figure 1: flowchart of participants included in main analyses. Supplementary Table 1: comparison of characteristics between participants who provided prenatal dietary data and those who did not. Supplementary Tables 2–5: associations of selected nutrients with PSQI scores from models removing covariates one by one to identify positive confounding variables.
