# Supplementary material for: Dietary Intake of Nutrients Involved in Serotonin and Melatonin Synthesis and Prenatal Maternal Sleep Quality and Affective Symptoms
Source: J Nutr Metab. 2024 Jul 8;2024:6611169. doi: 10.1155/2024/6611169 (PMC11250910; doi:10.1155/2024/6611169)
Supplement: Supplementary Materials — Supplementary Figure 1: flowchart of participants included in main analyses. Supplementary Table 1: comparison of characteristics between participants who provided prenatal dietary data and those who did not. Supplementary Tables 2–5: associations of selected nutrients with PSQI scores from models removing covariates one by one to identify positive confounding variables. [file 6611169.f1.docx]

Enrolled

N=326

Prenatal Dietary Data Available N=270

EPDS

N=261

PSWQ

N=259

Lost to follow-up/withdrawn: N=11

No dietary data collected: N=29

Miscarriage: N=5

Became Ineligible: N=11

PSQI

N=242

Supplementary Figure 1: Flowchart of Participants with Prenatal Dietary Data and Prenatal Sleep Quality, Depressive Symptom, or Anxiety Symptom Data.

*PSQI= Pittsburgh Sleep Quality Index, EPDS= Edinburgh Postnatal Depression Scale, PSWQ=Penn State Worry Questionnaire.*

| Supplementary Table 1. Comparison of Characteristics by Availability of Prenatal Dietary Data | | | | |
| --- | --- | --- | --- | --- |
|  | Sample Size^1^ | Diet data | No diet data | P-value^2^ |
| Race and Ethnicity, N (%) | 270/56 |  |  | 0.091 |
| NH White |  | 156 (57.78) | 24 (42.86) |  |
| NH Black |  | 63 (23.33) | 22 (39.29) |  |
| Hispanic |  | 29 (10.74) | 6 (10.71) |  |
| Asian and Other |  | 22 (8.15) | 4 (7.14) |  |
|  |  |  |  |  |
| Education, N (%) | 270/49 |  |  | 0.062 |
| ≤ High School Diploma |  | 95 (35.19) | 27 (55.10) |  |
| Some College |  | 39 (14.44) | 5 (10.20) |  |
| Bachelor’s Degree |  | 68 (25.19) | 10 (20.41) |  |
| Post Graduate Degree |  | 68 (25.19) | 7 (14.29) |  |
| Parity, N (%) | 270/47 |  |  | 0.443 |
| Nulliparous |  | 96 (35.56) | 14 (29.79) |  |
| Smoking During Pregnancy, N (%) | 261/44 | 18 (6.90) | 5 (11.36) | 0.349 |
| Yes |  |  |  |  |
| Trimester 2 Medication Use, N (%) |  |  |  |  |
| Sleeping Medication | 251/23 | 37 (14.74) | 1 (4.35) | 0.220 |
| SSRI | 161/12 | 12 (7.45) | 1 (8.33) | 1.000 |
| Psychiatric not SSRI | 161/12 | 4 (2.48) | 0 (0) | 1.000 |
| Trimester 3 Medication Use, N (%) |  |  |  |  |
| Sleeping Medication | 229/22 | 33 (14.41) | 3 (13.64) | 1.000 |
| SSRI | 154/12 | 14 (9.09) | 1 (8.33) | 1.000 |
| Psychiatric not SSRI | 154/12 | 4 (2.60) | 0 (0) | 1.000 |
| Age, years (mean, SD) | 270/56 | 29.17 (4.56) | 27.05 (5.12) | **0.002** |
| Physical Activity, Median (25^th^, 75^th^) |  |  |  |  |
| T2 MVPA met hours/week | 259/25 | 118.82  (72.62, 202.30) | 119.88  (84.35, 203.52) | 0.753 |
| T3 MVPA met hours/week | 251/24 | 103.08  (57.22, 185.50) | 121.71  (72.98, 251.30) | 0.264 |
| Early Pregnancy BMI^3^, Median (25^th^, 75^th^) | 270/48 | 26.26  (22.86, 32.28) | 29.89  (25.59, 33.83) | **0.030** |
| *^1^ Sample sizes are reported for participants with diet data/ participants without diet data*  *^2^P-values were generated from t-tests for normally distributed continuous variables, Kruskal Wallis tests for non-normal continuous variables, and chi square tests or Fisher’s exact tests for categorical variables*  *MVPA= Moderate to Vigorous Physical Activity, BMI = Body Mass Index, SSRI= Selective Serotonin Reuptake Inhibitor* | | | | |

| Supplementary Table 2. Associations of selected nutrients^1^ with Global PSQI score from 1) extended models^2^, 2) models removing each covariate (from the extended model) one by one to identify positive confounding variables^3^, and 3) models simultaneously removing all positive confounding variables | | | | |
| --- | --- | --- | --- | --- |
|  | EPA+DHA (N=238)^4^ | | Tryptophan (N=238)^4^ | |
| Models | Beta | 95% CI^5^ | Beta | 95% CI^5^ |
| Extended model^2^ | -0.92 | -1.79, -0.06 | 0.22 | -14.06, 14.50 |
| Remove percent of calories from fat | -0.90 | -1.58, -0.22 | - | - |
| Remove percent calories from carbohydrate | - | - | -1.51 | -16.15, 13.14 |
| Remove percent calories from protein | - | - | -0.20 | -0.05, 0.15 |
| Remove age | -0.86 | -1.61, -0.12 | -3.63 | -16.98, 9.73 |
| Remove body mass index | -0.85 | -1.68, -0.03 | -1.81 | -15.96, 12.34 |
| Remove race and ethnicity | -0.81 | -1.45, -0.17 | -5.76 | -19.22, 7.71 |
| Remove education | -0.84 | -1.58, -0.10 | 0.32 | -13.42, 14.05 |
| Remove parity | -0.85 | -1.65, -0.59 | -0.30 | -14.34, 13.75 |
| Remove moderate and vigorous physical activity (MVPA) metabolic equivalents of task (METs) | -1.60 | -3.32, 0.11 | 3.02 | -12.90, 18.93 |
| Remove smoking during pregnancy | -0.74 | -1.32, -0.16 | -3.32 | -17.06, 10.41 |
| Remove sleeping medication use | -1.14 | -1.96, -0.32 | 0.96 | -15.80, 17.71 |
| Simultaneous removal of positive confounding variables^6^ | -1.33 | -2.34, -0.33 | -9.03 | -16.91, -1.15 |
| *^1^ Selected nutrients included those that were statistically significantly associated with global PSQI score in minimally adjusted models, but attenuated to non-significance in extended models*  *^2^ The extended model adjusted for trimester, age, body mass index, race and ethnicity, education, parity, physical activity, smoking during pregnancy, sleeping medications, and macronutrient intake (i.e., percent of calories from fat for EPA+DHA models, and percent of calories from protein and carbohydrate for tryptophan models)*  *^3^ A positive confounding variable is defined as one that resulted in ≥10% strengthening of the effect estimate (i.e., in the negative direction) upon their individual removal from the extended model*  *^4^Sample size reflects participants with complete covariate data*  *^5^ 95% CIs were not calculated with bootstrapping*  *^6^ Positive confounding variables were identified to be MVPA and sleeping medication for the EPA+DHA and PSQI association, and percentage of calories from carbohydrate and protein, age, body mass index, race and ethnicity, parity, and smoking during pregnancy for the tryptophan and PSQI association* | | | | |

| Supplementary Table 3. Associations of tryptophan with PSQI sleep latency component score from 1) the extended model^1^ and 2) models removing each covariate (from the extended model) one by one to identify positive confounding variables^2^, N=243^3^ | | |
| --- | --- | --- |
| Models | Odds Ratio | 95% CI^4^ |
| Extended model | 0.91 | 0.83, 1.00 |
| Remove percent calories from carbohydrate | 0.91 | 0.83, 1.00 |
| Remove percent calories from protein | 0.94 | 0.87, 1.02 |
| Remove age | 0.90 | 0.83, 0.98 |
| Remove body mass index | 0.91 | 0.83, 1.00 |
| Remove race and ethnicity | 0.91 | 0.83, 0.99 |
| Remove education | 0.91 | 0.83, 0.99 |
| Remove parity | 0.91 | 0.83, 1.00 |
| Remove moderate and vigorous physical activity (MVPA) metabolic equivalents of task (METs) | 0.91 | 0.83, 1.00 |
| Remove smoking during pregnancy | 0.91 | 0.83, 1.00 |
| Remove sleeping medication use | 0.92 | 0.84, 1.02 |
| *^1^ The extended model adjusted for trimester, age, body mass index, race and ethnicity, education, parity, physical activity, smoking during pregnancy, sleeping medications and percent of calories from protein and carbohydrate*  *^2^ A positive confounding variable is defined as one that resulted in ≥10% strengthening of the effect estimate (i.e., in the negative direction) upon their individual removal from the extended model*  *^3^Sample size reflects participants with complete covariate data*  *^4^ 95% CIs were not calculated with bootstrapping* | | |

| Supplementary Table 4. Associations of selected nutrients^1^ with PSQI sleep duration component score from 1) extended models^2^ and 2) models removing each covariate (from the extended model) one by one to identify positive confounding variables^3^ | | | | |
| --- | --- | --- | --- | --- |
|  | EPA+DHA (N=244)^4^ | | Tryptophan (N=244)^4^ | |
| Models | Odds Ratio | 95% CI^5^ | Odds Ratio | 95% CI^5^ |
| Extended model^2^ | 0.83 | 0.55, 1.26 | 1.00 | 0.89, 1.12 |
| Remove percent of calories from fat | 0.87 | 0.60, 1.25 | - | - |
| Remove percent calories from carbohydrate | - | - | 0.99 | 0.88, 1.11 |
| Remove percent calories from protein | - | - | 1.02 | 0.93, 1.10 |
| Remove age | 0.81 | 0.53, 1.22 | 0.97 | 0.87, 1.07 |
| Remove body mass index | 0.83 | 0.56, 1.24 | 0.96 | 0.86, 1.08 |
| Remove race and ethnicity | 0.79 | 0.53, 1.19 | 0.95 | 0.84, 1.06 |
| Remove education | 0.82 | 0.54, 1.24 | 1.00 | 0.90, 1.13 |
| Remove parity | 0.80 | 0.54, 1.20 | 1.00 | 0.89, 1.12 |
| Remove moderate and vigorous physical activity (MVPA) metabolic equivalents of task (METs) | 0.80 | 0.53, 1.21 | 0.99 | 0.88, 1.11 |
| Remove smoking during pregnancy | 0.82 | 0.55, 1.25 | 1.00 | 0.89, 1.12 |
| Remove sleeping medication use | 0.84 | 0.56, 1.26 | 1.00 | 0.89, 1.12 |
| *^1^ Selected nutrients included those that were statistically significantly associated with global PSQI score in minimally adjusted models, but attenuated to non-significance in extended models*  *^2^ The extended model adjusted for trimester, age, body mass index, race and ethnicity, education, parity, physical activity, smoking during pregnancy, sleeping medications and macronutrient intake (i.e., percent of calories from fat for EPA+DHA models, and percent of calories from protein and carbohydrate for tryptophan models)*  *^3^ A positive confounding variable is defined as one that resulted in ≥10% strengthening of the effect estimate (i.e., in the negative direction) upon their individual removal from the extended model*  *^4^Sample size reflects participants with complete covariate data*  *^5^ 95% CIs were not calculated with bootstrapping* | | | | |

| Supplementary Table 5. Associations of selected nutrients^1^ with PSQI sleep disturbances component score from 1) extended models^2^ and 2) models removing each covariate (from the extended model) one by one to identify positive confounding variables^3^ | | | | |
| --- | --- | --- | --- | --- |
|  | EPA+DHA (N=243)^4^ | | Tryptophan (N=243)^4^ | |
| Models | Odds Ratio | 95% CI^5^ | Odds Ratio | 95% CI^5^ |
| Extended model^2^ | 0.79 | 0.53, 1.19 | 0.93 | 0.85, 1.03 |
| Remove percent of calories from fat | 0.72 | 0.50, 1.04 | - | - |
| Remove percent calories from carbohydrate | - | - | 0.92 | 0.84, 1.01 |
| Remove percent calories from protein | - | - | 0.98 | 0.90, 1.07 |
| Remove age | 0.78 | 0.52, 1.17 | 0.92 | 0.84, 1.01 |
| Remove body mass index | 0.79 | 0.53, 1.19 | 0.93 | 0.84, 1.02 |
| Remove race and ethnicity | 0.73 | 0.50, 1.08 | 0.91 | 0.83, 1.00 |
| Remove education | 0.80 | 0.54, 1.20 | 0.93 | 0.85, 1.03 |
| Remove parity | 0.81 | 0.54, 1.22 | 0.93 | 0.85, 1.03 |
| Remove moderate and vigorous physical activity (MVPA) metabolic equivalents of task (METs) | 0.76 | 0.50, 1.14 | 0.93 | 0.85, 1.03 |
| Remove smoking during pregnancy | 0.79 | 0.53, 1.18 | 0.93 | 0.84, 1.03 |
| Remove sleeping medication use | 0.78 | 0.52, 1.17 | 0.94 | 0.85, 1.04 |
| *^1^ Selected nutrients included those that were statistically significantly associated with global PSQI score in minimally adjusted models, but attenuated to non-significance in extended models*  *^2^ The extended model adjusted for trimester, age, body mass index, race and ethnicity, education, parity, physical activity, smoking during pregnancy, sleeping medications and macronutrient intake (i.e., percent of calories from fat for EPA+DHA models, and percent of calories from protein and carbohydrate for tryptophan models)*  *^3^ A positive confounding variable is defined as one that resulted in ≥10% strengthening of the effect estimate (i.e., in the negative direction) upon their individual removal from the extended model*  *^4^Sample size reflects participants with complete covariate data*  *^5^ 95% CIs were not calculated with bootstrapping* | | | | |
